# Assessing Molecular Diversity in Native and Introduced Populations of Red Wood Ant *Formica paralugubris*

**DOI:** 10.3390/ani12223165

**Published:** 2022-11-16

**Authors:** Alberto Masoni, Andrea Coppi, Paride Balzani, Filippo Frizzi, Renato Fani, Marco Zaccaroni, Giacomo Santini

**Affiliations:** 1 Department of Biology, University of Florence, 50019 Florence, Italy; 2 Faculty of Fisheries and Protection of Waters, South Bohemian Research Center of Aquaculture and Biodiversity of Hydrocenoses, University of South Bohemia in České Budějovice, 38925 Vodňany, Czech Republic

**Keywords:** red wood ants, Foreste Casentinesi National Park, introduced species, AFLP, genetic diversity

## Abstract

**Simple Summary:**

Red wood ants are ecologically dominant ant species that play key roles in boreal forest ecosystems, where they greatly influence the habitat dynamics with their predatory activity. During the last century, they were largely employed as biocontrol agents in Italy against forest pests, and thousands of nests were transplanted from the Alps to the Apennines for this aim. We compared genetic variability and structure of native and introduced populations of *F. paralugubris* by AFLP assay and found that it was higher in the introduced populations, while native ones showed a higher diversity between nests. Overall, the genetic structure was dominated by among-worker variation regardless of different grouping arrangement (Alps vs. Apennine, native vs. introduced).

**Abstract:**

The *Formica rufa* group comprises several ant species which are collectively referred to as “red wood ants” and play key roles in boreal forest ecosystems, where they are ecologically dominant and greatly influence habitat dynamics. Owing to their intense predatory activity, some of these species are used as biocontrol agents against several forest insect pests and for this aim in Italy, nearly 6000 ant nests were introduced from their native areas in the Alps to several Appeninic sites during the last century. In this work, we assessed and compared the genetic variability and structure of native and introduced populations of *F. paralugubris*, thus evaluating the extent of genetic drift that may have occurred since the time of introduction, using amplified fragment length polymorphism (AFLP) markers. PCR amplification with a fam_EcoRI-TAC/MseI-ATG primers combination produced a total of 147 scorable bands, with 17 identified as outlier loci. The genetic variation was higher in the introduced population compared to the native ones that, on the other hand, showed a higher diversity between nests. AMOVA results clearly pointed out that the overall genetic structure was dominated by among-worker variation, considering all populations, the Alpine vs. Apennine groups and the comparison among native and related introduced populations (all ranging between 77.84% and 79.84%). Genetic analyses unveiled the existence of six main different groups that do not entirely mirror their geographic subdivision, pointing towards a wide admixture between populations, but, at the same time, rapid diversification of some Apennine populations. Future studies based on high-throughput genomic methods are needed to obtain a thorough understanding of the effects of environmental pressure on the genetic structure and mating system of these populations.

## 1. Introduction

Red wood ants (RWA) are ecologically dominant species native of boreal forests of Central and Northern Europe [1]. They belong to the *Formica rufa* Palearctic complex, which in Western Europe comprises at least six species: *F. rufa* (Linneus, 1758), *F. aquilonia* (Yarrow, 1955), *F. lugubris* (Zetterstedt, 1838), *F. paralugubris* (Seifert, 1996), *F. polyctena* (Foerster, 1850) and *F. pratensis* (Retzius, 1783). All these species are characterised by the ability to build large aboveground nest mounds, and by the red and black coloration of their bodies. From the ecological point of view, RWA are keystone species, and they deeply impact the functioning of their forest ecosystems across multiple trophic levels [2]. These species influence the dynamics of arthropod communities through predation and competition [3], the structure of plant and lichen communities through their action on aphids, parasites and herbivores or propagule dispersion [4,5] and, ultimately, they affect nutrient cycling and soil functioning [6,7]. Despite the key role and abundance in most of their distribution range, the conservation status of this species is raising increasing concerns as there is evidence of local decline and even local extinction [8,9].

Owing to their intense predatory activity, some RWA species have been employed as biocontrol agents against several forest insect pests [10]. For this purpose, in Italy and Germany, nests of these species were transplanted from their original areas to other sites where they were formerly absent [11]. Between 1958 and 1972, more than 6000 nests of *F. lugubris/paralugubris*, *F. polyctena* and *F. aquilonia* were repeatedly transferred from the Alps to the Apennines and other Italian mountainous areas [12]. These introductions were carried out without considering the possible risks caused by the numerical reduction of the native populations, nor their possible negative impact on the newly occupied ecosystems, and it is worth mentioning that nowadays this practice is forbidden [13]. While in some cases the introductions resulted in viable populations that started to expand, actively preying upon the arthropod fauna in the newly occupied areas, other attempts failed [12,13]. The status of most of these introduced populations, similar to that of the native populations in the Alps, is unknown, and certainly calls for further studies [9]. Studying the ecology of these introduced populations is scientifically relevant, as they are a sort of a unique long-term ecological experiment that can provide important information on community dynamics, the effect of the introduction of dominant species, and population genetics.

*Formica paralugubris* was one of the most frequently introduced species in the Italian peninsula, but also to Canada [14,15] and was described for the first time as a sibling species of *F. lugubris* by Seifert [16]. Since its description, autochthonous populations of this species were discovered in the Pyrenees, Alps and the Jura mountains, at elevations ranging from 600 m to 2200 m asl [17]. *Formica paralugubris* can form huge supercolonies, composed of tens to hundreds of interconnected nests that may cover areas of over 0.5 km^2^ [18,19], where they outcompete other ant species and affect other arthropod communities [20]. Each nest may contain hundreds of reproductive queens and two different reproductive strategies have been described. In one case, sexuals mate and remain within their natal colony, while in the other they perform mating flights that ensure long-distance dispersal [21]. The two strategies have a deep impact on the genetic structure of colonies, nest networks and relatedness within the nest. In general, long-distance dispersal through mating flights is rare, while budding is by far the most common colony reproduction mechanism. As a consequence, nearby nests in a supercolony are usually genetically closer than distant ones [22].

In the present study, we investigated the genetic variability in two native and four introduced populations (two from each native population) of *F. paralugubris*, using amplified fragment length polymorphism (AFLP) analysis. AFLPs are a PCR-based highly replicable dominant marker that allows rapid screening of genetic diversity and intraspecific variation without a priori sequence knowledge and at a low cost [23]. Our goal was to compare the genetic structure of native and introduced populations and evaluate the extent of genetic drift that may have occurred since the time of introduction 70 years ago. Furthermore, these local populations are reproductively isolated from those in the Alps and subjected to different selection pressures than those experienced by the native populations, given the lower latitude and altitude of their habitats. Based on these premises, we hypothesised that (i) the introduced populations show signatures of divergence from their native population while retaining some similarity among them; that (ii) the genetic variability is higher in the native Alpine populations than in those introduced in the Apennines. Our results will be a blueprint for future studies based on high-throughput genomic methods applied to selected nest samples; this will provide sufficient power for a thorough understanding of the effects of environmental pressure on the genetic structure, and mating system of these ant populations.

## 2. Materials and Method

### 2.1. Sampling Site

**Figure 1:**
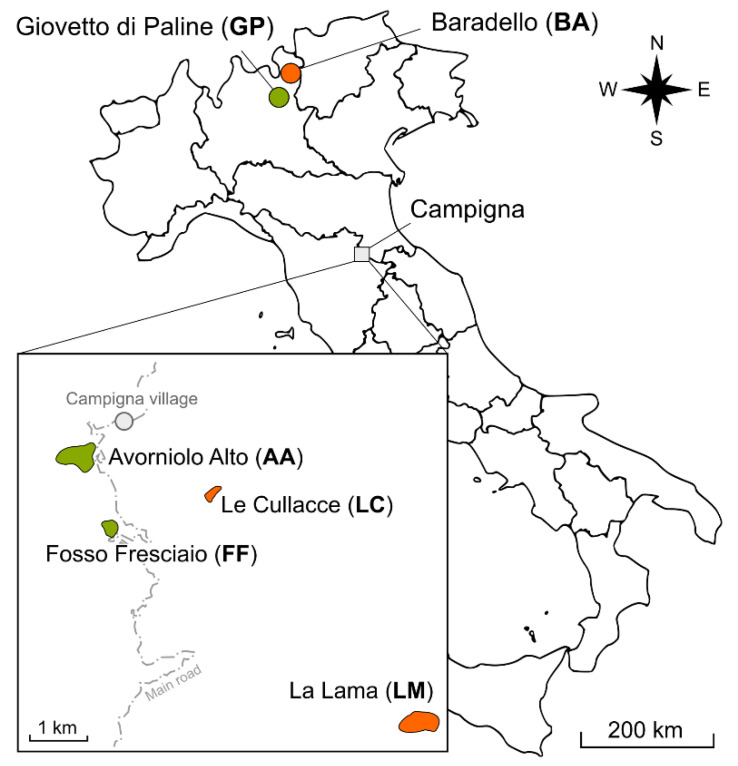
Geographic location of *F. paralugubris* populations investigated in this study.

*Formica paralugubris* ants were collected from six populations—two native from the Alps and four introduced in the Apennine (Figure 1)—from June to August 2019. The Alpine populations were from Giovetto di Paline Nature Reserve (abbreviated as GP, 45°57′57″ N, 10°7′48″ E) and Baradello (abbreviated BA, 46°08′40″ N, 10°10′08″ E). We identified and sampled the populations located in the same areas reported as the origin of the introduced nests, and we assumed they are the descendants of the original ones. The introduced populations were from the Foreste Casentinesi, Monte Falterona and Campigna National Park, where nests of this species had been repeatedly introduced between 1958 and 1964. These populations were from the following locations: Avorniolo Alto (abbreviated as AA, 43°52′03″ N, 11°44′15″ E), Fosso Fresciaio (abbreviated FF, 43°51′23″ N, 11°44′36″ E), Le Cullacce (abbreviated LC, 43°51′42″ N, 11°45′50″ E) and La Lama (abbreviated LM, 43°49′36″ N, 11°48′24″ E). Populations from AA and FF originated from nests collected at GP, while the ones at LC and LM were from nests collected at BA. The Alpine sites were characterised by mixed forest, composed of a dominant conifer species, the Norway spruce (*Picea abies*, H.Karst., 1881), and beech (*Fagus sylvatica*, Linneaus, 1753). LC and FF were also characterised by mixed forest, but the local dominant conifer species is the silver fir (*Abies alba*, Miller, 1759), while AA and LM are covered by almost pure silver fir stands. More details on the ecology of the introduced populations and the history of their introduction can be found in [11,13].

For each population, we selected ten nests located at least 50 m apart, and ten ants were collected from the surface of each nest, for a total of 600 workers. The geographic position of the sampled nests was recorded by a GPS locator (Garmin eTrex^®^ 10, accuracy ~3 m, Garmin, Olathe, KS, USA). Ants were stored in plastic test tubes (8 mL volume) filled with pure reagent-grade ethanol. All samples were transferred to the laboratory within 24 h from collection and stored at −80 °C for genetic analysis.

One individual per nest at BA and LM was identified as F. paralugubris following the molecular method developed by Bernasconi et al. [24], while species identity of ants collected in the other sites had been corroborated in previous studies [15,25].

### 2.2. AFLP Fingerprinting

Genomic DNA was extracted from a single adult thorax (10 individuals per nest), with a modified Chelex method [26]. Thorax without legs was ground into an Eppendorf plastic tube (1.5 µL) filled with 100 µL of 5% Chelex (BioRad, Hercules, CA, USA) solution (5 mg Chelex 100 resin in 50 mL ddH_2_O). The sample was gently stirred and incubated at 56 °C for 4 h, adding 4 µL of Proteinase K. After centrifugation at 13,000 for 3 min, the upper aqueous supernatant was precipitated with 100% ethanol and 3 M sodium acetate, pH 5.2. This mixture was incubated at −20 °C for 20 min and then centrifuged at maximum speed for 10 min. The resulting pellet was washed by adding 70% ethanol and then centrifuged for 10 min at maximum speed. The supernatant was discarded and the pellet was airdried, resuspended in 30 µL of DNase-free water (Invitrogen, Waltham, MA, USA) and used as a template for PCR amplification. The extracted DNA was quantified using a Qubit 4 fluorometer (Invitrogen) with the dsDNA HS Assay kit (Invitrogen™ Q33231).

An analysis of AFLP divergence was performed following the procedure described by Coppi et al. [27]. The quality of AFLP profiles, and hence of the DNA extraction method, was preliminarily tested on 24 samples randomly selected from the 6 populations, evaluating six primer pair combinations. The fam_EcoRI-CTA/MseI-TTA combination was selected because of its highly comparable results across all the samples (in terms of good PCR products as well as of the number and size of the peaks obtained). Analysis of the AFLP profiles obtained by running capillary electrophoresis with the Applied Biosystems 3130xl platform was performed with GeneMarker v1.5 (SoftGenetics LLC, State College, PA, USA). A cut-off value, fixed at 5% of the maximum profile showed in the chromatograms, was determined after the analysis of replicate samples (reproducibility of the data was assessed by replicating 20 samples that were marked as duplicated and compared with the rest of the dataset in GeneMarker), considering only bands present in all the replicates. AFLP loci under selection (outliers) were screened using a Bayesian probability approach implemented in BayeScan v.2.01 [28]. The posterior probability of a given locus under selection was estimated, assuming that the locus frequencies within a population follow a multivariate β-distribution as a function of the multilocus fixation index value and of the average of locus frequencies of each locus between populations [29,30]. The analysis was set according to the software manual, considering 20 pilot runs with a length of 10,000 iterations each. The mean number of outlier loci was determined for each population.

### 2.3. AFLP Analysis

#### 2.3.1. Genetic Diversity

Since AFLP are dominant markers, the presence or absence of every single fragment (100–2000 bp) was scored in each sample and coded by 1 or 0, creating a binary data matrix used to evaluate the within-population genetic variation as percentage of polymorphic loci (PL%), standard Nei’s measure of genetic diversity (“h”) as “average gene diversity over loci” [31] and Shannon’s information index (“I”) [32] for all populations in POPGENE v.1.32 [33].

The analysis of molecular variance (AMOVA) was performed using the ARLEQUIN v. 2.000 [34] to determine the partitioning of the overall genetic variation among all populations, between the Alpine and Apennine groups, and between the native populations and their related introduced ones, considering different hierarchical levels: between populations, among nests within populations and among workers. The analyses were performed separately, considering different hypothetical population groupings tested in terms of the variance components and the percentage of total expressed variation. Genetic diversity among nests within each population was assessed evaluating the fixation index (F_ST-POP_), as difference in the allele frequency between nests.

#### 2.3.2. Genetic Distance and Structure

A pairwise distances matrix among workers was computed following the Tamura–Nei method [35] and then a clustering analysis among nests, based on unweighted pair-group method with arithmetic means (UPGMA), was carried out in POPGENE and MEGA v.10.2.4 [36].

The analysis of population structures was performed following a model-based Bayesian clustering method implemented in STRUCTURE v.2.3.4 [37]. An admixture and shared allele frequency model was used to determine the number of clusters (K), assumed to be in the range between 2 and 12, with 10 replicate runs for each potential group. For each run, the initial burn-in period was set to 20,000 with 200,000 MCMC (Markov chain Monte Carlo) iterations, with no prior information on the origin of individuals. The best fit for the number of clusters, K, was determined using the Evanno method [38], as implemented in STRUCTURE HARVESTER [39]. STRUCTURE results were then elaborated using the R\pophelper package to align cluster assignments across replicate analyses and produce visual representations of cluster assignments.

## 3. Results

The AFLP dataset resulted from the analysis of 534 samples (Table 1), since the other 66 were lost (two whole nests at BA8 and LC10) because of bad quality amplification. The selected primers fam_EcoRI-TAC/MseI-ATG produced a total of 147 scorable bands with molecular weights ranging from approximately 40 to 550 base pairs. The BayeScan analysis identified 17 outlier loci that had a posterior probability higher than 0.8 (at a threshold of log10 PO ranging from 0.5 and 3), representing the 5% of all analysed loci. The highest locus diversity was found in LC, LM and AA with a mean number of outlier loci of 6.88, 6.34 and 6.26, respectively, whereas the lowest number was found at BA (3.21). GP and FF showed intermediate values amounting to 4.73 and 4.86, respectively. For the sake of clarity, no locus was excluded in any of the analyses.

The percentage of polymorphic loci (PL%) among population ranged from a maximum of 90.48% (LC) to a minimum of 51.7% (BA). A summary of all the diversity measured is reported in Table 1. Genetic diversity (h) varied among populations and the highest h values were observed in the two introduced populations, AA and LC. If populations were divided according to a latitudinal gradient, the Apennine ones had higher values of diversity compared to their Alpine counterpart. A comparable trend can be also observed for either for PL%, and I values.

When we considered the population differentiation (measured by F_ST-POP_, h and I), it appeared that the two native populations had the lowest values of genetic diversity (Table 1) but the highest among their nests. AMOVA showed significant differentiation among all populations and between latitudinal (Alpine vs. Apennine) groups (F_ST_ = 0.206, *p* < 0.001; F_ST_ = 0.221, *p* < 0.001, respectively). Nearly the same situation was found between the native populations and their related introduced ones (F_ST_ = 0.217, *p* < 0.001, F_ST_ = 0.218, *p* < 0.001, respectively, for BA vs. LC + LM and GP vs. AA + FF). A genetic structure dominated by among-worker variation (see Table 2) was evident at all levels and did not correspond to comparable among-nets variations.

**Figure 2:**
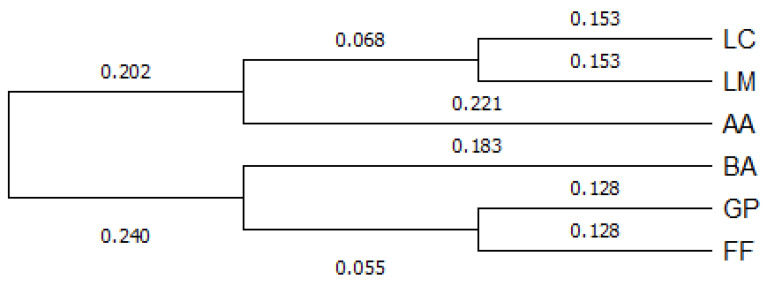
UPGMA dendrogram of investigated populations based. Studied populations: LC, Le Cullacce; LM, La Lama; AA, Avorniolo Alto; BA, Baradello; GP, Giovetto di Paline; FF, Fosso Fresciaio.

The UPGMA-based dendrogram (Figure 2) showed that the six populations clustered into two major groups: one comprising the native Alpine populations and FF, and one including the other Apenninic populations (LC, LM and AA). When looking at the second cluster, the three Apennine populations clustered together, with LC and LM very close to each other.

**Figure 3:**
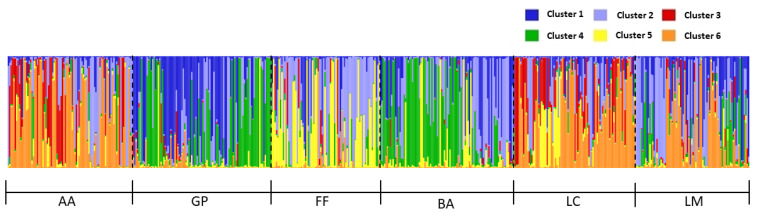
STRUCTURE plot of the 534 worker genotypes. Each individual is represented by a vertical line; the genetic clusters identified are numbered from 1 to 6 and marked with different colours. Population affiliation is also indicated: LC, Le Cullacce; LM, La Lama; AA, Avorniolo Alto; BA, Baradello; GP, Giovetto di Paline; FF, Fosso Fresciaio.

The population genetic kinship obtained with the UPGMA-based tree was subsequently confirmed by STRUCTURE results (Figure 3). Worker genotypes were assigned to a cluster with a probability >0.6. The optimum number of populations, K, estimated (Figure S1) according to the Evanno method, was six. This suggests the occurrence of six populations (i.e., gene pools) which were nonetheless clearly admixed and do not correspond to the geographic origin of the samples. The two Alpine populations (GP and BA) were highly similar and quite different from the others. FF population was relatively close to the Alpine ones, due to the sharing of several alleles, especially with BA, but also showed some distinctiveness compared to the other Apennine populations. AA and LC seemed quite differentiated from their population of origin and similar to each other, while LM showed all the six gene pools highly admixed.

## 4. Discussion and Conclusions

This study assessed the variation of genetic diversity and structure across *F. paralugubris* populations translocated to the Apennines and as compared with their Alpine populations of origin 70 years after the introduction. We successfully used an AFLP assay for detecting overall differentiation among and within populations, on the basis of 147 loci.

About 5% of the identified loci can be considered outliers and although it was impossible to directly associate them specifically with any of the surveyed populations, it is important to note that, overall, they were more numerous in the introduced ones. The different pedoclimatic conditions, altitude, average temperature and vegetation between Alps and Apennines may account for the observed difference in outlier frequency and expression [40], although this finding certainly requires further study.

Our results also confirmed the existence of considerable genetic admixture in all the surveyed populations, which was expected, based on the observations on the reproductive behaviour reported by Frizzi et al. [41] and the findings on other European *F. paralugubris* populations as well as other RWA species [19]. The low genetic variance among nests (8% of total) suggests gene flow between them and is also consistent with budding reproductive behaviour, characterised by mating occurring in close proximity or even within the natal nest [20]. On the contrary, the high intra-nest genetic variance, which amounts to nearly 80% of the total observed variation, confirms that multiple unrelated or weakly unrelated queens may inhabit the same nest [22]. Finally, the low among-populations variability (12% of total variance) can be explained by considering that all the colonies belong to the same native macro area in the Alps, and have been randomly separated nearly seven decades ago, which is a short time in terms of natural selection [42]. If we compare the genetic variance between Apennine and Alpine populations, we see nearly the same trend, with the major proportion of variance being partitioned among workers within each population (78%). However, we have to point out that, in this respect, the Alpine populations were under-represented, since only 20 nests were analysed, as opposed to the 40 from the Apennines. Another important point to consider is that we cannot be completely sure that the colonies we sampled as native populations are really the direct descendants of the ants transplanted 70 years ago. We sampled exactly the same areas, but we could not exclude possible supercolony replacement events during the last decades.

Contrary to our expectation, the introduced LM, LC, and AA had the greatest genetic diversity, while the lowest values were detected in the native Alpine populations. These were characterised by a low number of polymorphic loci and the highest values of F_ST-POP_ genetic distance among nests. This finding suggests that the gene pool of these populations might have undergone a reduction through the years, presumably as a consequence of demographic decline [43]. This point deserves careful attention and cannot be overlooked. Paradoxically, while the populations introduced to the Apennines are expanding [13], the status of the populations in the Alps is poorly known, and there are no indications of whether they are stable, increasing, or decreasing [9]. It is likely that the massive collection of nests carried out during the last century for transplantation [12] could have caused a non-negligible reduction of their size and, hence, genetic diversity [44]. However, it should be kept in mind that we have no information on the genetic structure and variability of the original Alpine populations at the time of nest collection, making further speculations difficult.

As for the populations in the Apennines, there is no doubt that the new habitat differed in many ways from that in the original locations in the Alps, both as composition of the forest stands (i.e., presence of silver fir instead of Norway spruce) and, more importantly, in climate, being located more than 250 km south of the Alps. The concurrent action of these factors could have imposed local selective pressures underlying the observed changes. Moreover, we also know that most of the original nest mounds introduced (20 at AA, 19 at FF, 27 and 20 at LM and LC, respectively) had disappeared a few years later, and in nearly all areas the populations had reduced to three to six mounds [45]. We do not know exactly whether this was due to colony death, followed by selection and bottleneck effect, or it resulted from their fusion into few larger ones. The initial drastic reduction in nests number may have affected the genetic pool of the new populations in two opposite ways. If the reduction was due to nest aggregation, the initial genetic diversity should have been retained, at least initially. Alternatively, if the reduction was due to colony death, genetic drift would have occurred. The high genetic variability among Apennine populations detected by our analyses suggests an initial aggregation dynamic. However, further studies based on different genetic markers (e.g., single nucleotide polymorphisms, SNPs) able to infer colony structure, mating system and overall diversity are needed to fully address all these issues [46].

The population genetic makeup obtained with STRUCTURE suggests the existence of six strongly admixed groups that do not correspond to the geographic origin. Admixture included elements from both the Apennine and the Alps, though AA, LC, LM and FF were seemingly different from their native ones. One point that needs to be carefully considered, however, is that the populations introduced to the Apennines 70 years ago may represent an important source of genetic diversity. Considering the low genetic variability of the native populations and increasing evidence of local declines and extinction in several parts of the species range, Alps included, these findings may have relevant implications for the conservation of RWA. Further studies are needed to better assess the status of the Alpine populations of *F. paralugubris*, while transplanted populations should be preserved through specific conservation policies and plans.

Finally, AFLP analysis turned out to be an efficient and cost-effective tool for assessing genetic diversity and variation in these populations, at least for a first screening based on population genetic variance. Compared to other markers such as RAPD (random amplified polymorphic DNA) and SSR (short sequence repeat) based on a polymerase chain reaction (PCR) step, AFLP can rapidly generate a great number of polymorphisms with a high number of assayed loci in virtually any organism, being often used for diversity and population genetics studies in ants and other insects [47,48,49]. The recent sequencing of the first RWA genome by Nouhaud et al. [50] opened up the prospect of using multiple next generation sequencing (NGS) approaches based on SNPs detection for population and phylogenetic studies of this ant group. For example, Portinha et al. [51] performed whole-genome resequencing of several workers to infer divergence histories among heterospecific populations of *F. polyctena* and *F. aquilonia*. As far as our populations are concerned, a double-digest restriction-site-associated DNA sequencing (ddRADseq) approach could be a powerful tool to compare the genetic variation, structure and mating system across populations [52,53] along with genome-wide association studies (GWAS) focused on the identification of the genes that are actively selected by the environmental factors and integrating phenotypic information [54].

In conclusion, this study provides the first information about variation in genetic diversity of native and introduced populations of *F. paralugubris* in Italy. The results may not only assist the conservation of native RWA populations but also better our understanding of the dynamics experienced by ant species when transplanted outside their distribution range.

## Figures and Tables

**Table 1:** Genetic diversity traits among populations: effective number of nests (*N_N_*) and of workers (*N_W_*) analysed; number of polymorphic loci (NPL) percentage of polymorphic loci (PL%); Nei’s genetic diversity (h, ±S.E.); fixation index (F_ST-POP_ ± S.E) calculated among nests of the same population Shannon’s information index (I, ±S.E.).

Population		locality	N_N_	N_w_	NPL	PL(%)	F_ST-POP_	h	I
GP	Native	Alps, Giovetto Paline	10	100	99	67.35	0.207 ± 0.003	0.104 ± 0.007	0.160 ± 0.020
AA	Transplanted	Apennine, Avorniolo	10	100	115	78.23	0.157 ± 0.006	0.184 ± 0.014	0.235 ± 0.027
FF	Transplanted	Apennine, Fosso Fresciaio	10	75	100	68.03	0.208 ± 0.005	0.104 ± 0.01	0.155 ± 0.023
									
BA	Native	Alps, Baradello	9	90	76	51.70	0.208 ± 0.002	0.102 ± 0.004	0.139 ± 0.021
LC	Transplanted	Apennine, Le Cullacce	9	87	133	90.48	0.147 ± 0.006	0.205 ± 0.011	0.231 ± 0.022
LM	Transplanted	Apennine, La Lama	10	82	118	80.71	0.183 ± 0.006	0.191 ± 0.012	0.199 ± 0.021

**Table 2:** Partitioning of genetic variation. AMOVA was performed, testing a four groupings scenario to test the differentiation between 534 individual samples from 6 populations. The table shows: degrees of freedom (df), sum of squared deviations, estimated variance components, percentages of total variance contributed by each component, three different fixation indexes and the probability of obtaining a more extreme component estimate by chance alone (P).

Source of Variation		df	Sum of Squares	Variance Components	% Variation	Fix.Index	*p*-Values
All population							
	Among populations	5	728.16	1.56	12.15	F_CT_ = 0.121	<0.001
	Among nests within populations	43	878.42	1.02	8.05	F_SC_ = 0.091	<0.001
	Among workers	435	4465.26	10.26	79.84	F_ST_ = 0.201	<0.001
	Total	483	6071.87	12.85	100		
Alpine vs. Apennine							
	Among groups	1	294.21	1.13	8.6	F_CT_ = 0.085	<0.05
	Among nests within groups	47	1312.33	1.78	13.56	F_SC_ = 0.148	<0.05
	Among workers	435	4465.22	10.26	77.84	F_ST_ = 0.221	<0.001
	Total	483	6071.81	13.17	100		
BA vs. (LC + LM)							
	Among groups	1	182.21	1.23	9.23	F_CT_ = 0.092	<0.001
	Among nests within groups	28	714.33	1.68	12.57	F_SC_ = 0.138	<0.001
	Among workers	239	2504.91	10.48	78.20	F_ST_ = 0.217	<0.001
	Total	268	3401.45		100		
GP vs. (AA + FF)							
	Among groups	1	144.39	0.94	7.65	F_CT_ = 0.076	<0.001
	Among nests within groups	28	701.93	1.75	14.20	F_SC_ = 0.153	<0.001
	Among workers	235	2269.92	9.65	78.15	F_ST_ = 0.218	<0.001
	Total	264	3116.24	12.35	100		

## Data Availability

Data will be made available on request.

## Supplementary Materials

The following supporting information can be downloaded at: https://www.mdpi.com/article/10.3390/ani12223165/s1, Figure S1: Plot of Delta-K values according to Evanno method for clusters identification.
