# Supplementary material for: Assessing Molecular Diversity in Native and Introduced Populations of Red Wood Ant Formica paralugubris
Source: Animals (Basel). 2022 Nov 16;12(22):3165. doi: 10.3390/ani12223165 (PMC9687034; doi:10.3390/ani12223165)
Supplement: Supplementary file 1 [file animals-12-03165-s001.zip › animals-1975378-supplementary.pdf]

Supplementary Materials

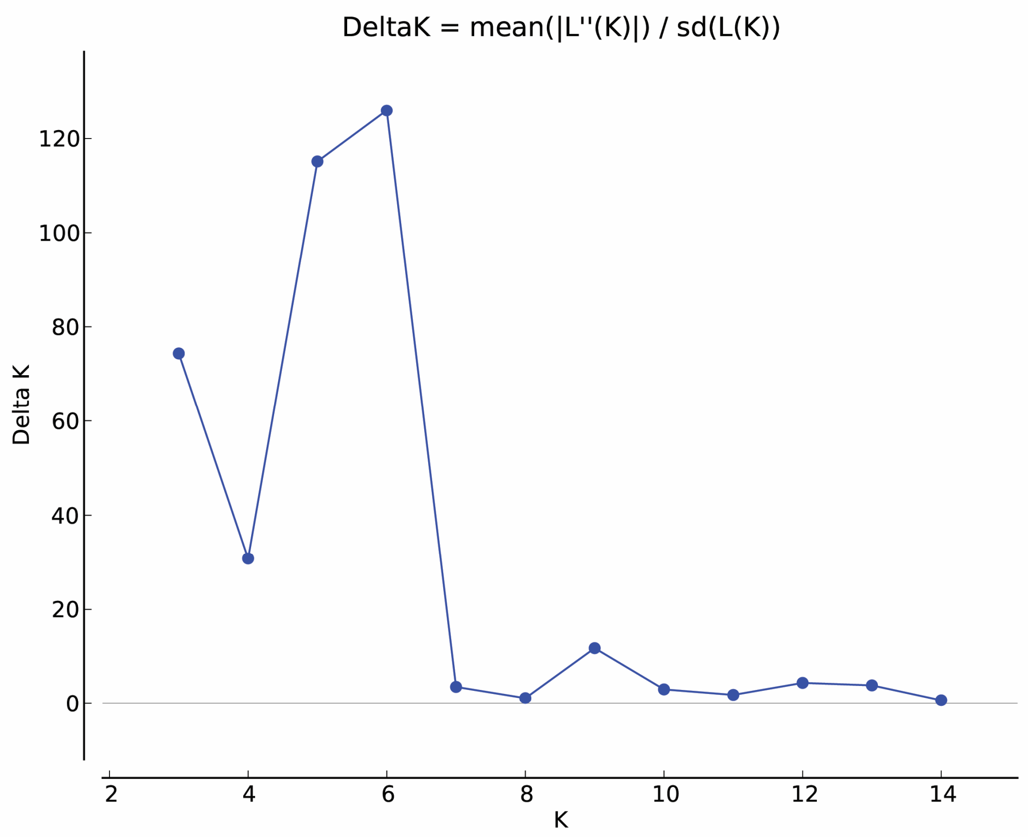

Figure S1: Plot of Delta-K values according to Evanno method for clusters identification.
